# Pattern Recognition Receptor Polymorphisms as Predictors of Oxaliplatin Benefit in Colorectal Cancer

**DOI:** 10.1093/jnci/djy215

**Published:** 2019-01-14

**Authors:** Victoria Gray, Sarah Briggs, Claire Palles, Emma Jaeger, Timothy Iveson, Rachel Kerr, Mark P Saunders, James Paul, Andrea Harkin, John McQueen, Matthew G Summers, Elaine Johnstone, Haitao Wang, Laura Gatcombe, Timothy S Maughan, Richard Kaplan, Valentina Escott-Price, Nada A Al-Tassan, Brian F Meyer, Salma M Wakil, Richard S Houlston, Jeremy P Cheadle, Ian Tomlinson, David N Church

**Affiliations:** See the Notes section for the full list of authors’ affiliations

## Abstract

**Background:**

Constitutional loss of function (LOF) single nucleotide polymorphisms (SNPs) in pattern recognition receptors *FPR1*, *TLR3*, and *TLR4* have previously been reported to predict oxaliplatin benefit in colorectal cancer. Confirmation of this association could substantially improve patient stratification.

**Methods:**

We performed a retrospective biomarker analysis of the Short Course in Oncology Therapy (SCOT) and COIN/COIN-B trials. Participant status for LOF variants in *FPR1* (rs867228), *TLR3* (rs3775291), and *TLR4* (rs4986790/rs4986791) was determined by genotyping array or genotype imputation. Associations between LOF variants and disease-free survival (DFS) and overall survival (OS) were analyzed by Cox regression, adjusted for confounders, using additive, dominant, and recessive genetic models. All statistical tests were two-sided.

**Results:**

Our validation study populations included 2929 and 1948 patients in the SCOT and COIN/COIN-B cohorts, respectively, of whom 2728 and 1672 patients had functional status of all three SNPs determined. We found no evidence of an association between any SNP and DFS in the SCOT cohort, or with OS in either cohort, irrespective of the type of model used. This included models for which an association was previously reported for rs867228 (recessive model, multivariable-adjusted hazard ratio [HR] for DFS in SCOT = 1.19, 95% confidence interval [CI] = 0.99 to 1.45, *P* = .07; HR for OS in COIN/COIN-B = 0.92, 95% CI = 0.63 to 1.34, *P* = .66), and rs4986790 (dominant model, multivariable-adjusted HR for DFS in SCOT = 0.86, 95% CI = 0.65 to 1.13, *P* = .27; HR for OS in COIN/COIN-B = 1.08, 95% CI = 0.90 to 1.31, *P* = .40).

**Conclusion:**

In this prespecified analysis of two large clinical trials, we found no evidence that constitutional LOF SNPs in *FPR1*, *TLR3*, or *TLR4* are associated with differential benefit from oxaliplatin. Our results suggest these SNPs are unlikely to be clinically useful biomarkers.

The antitumor immune response is an important determinant of clinical outcome in colorectal cancer (CRC). To date, attention has primarily focused on the role of the adaptive immune system, and particularly the T-cell response, the increasing intensity of which correlates with reduced recurrence in early-stage CRC (1,2). Although the influence of the innate immune system to clinical outcome is less well understood, several studies have suggested that this may also exert a meaningful antitumor effect through the recognition of endogenous ligands presented by dying cells (3–7). This effect has been reported to be especially relevant in the context of cell death induced by anthracyclines and oxaliplatin (3–5), an analog of cisplatin used commonly in the systemic therapy of CRC (8). Pattern recognition receptors present endogenous ligands to macrophages and as such are essential components of the innate immune response (9). Constitutional variants in several genes encoding these proteins have been shown to alter the innate immune response to systemic infection (10). Recently, polymorphisms that result in putative loss of function (LOF) alterations in pattern recognition receptor genes have also been reported to influence benefit from anthracycline and oxaliplatin chemotherapy (4–7). These variants, which affect *FPR1* [rs867228: c.1037A>C, p.Glu346Ala, where Ala is the LOF allele (11)], *TLR3* [rs3775291: c.1234C>T, p.Leu412Phe, where Phe is the LOF allele (12)], and *TLR4* [rs4986790: c.896A>G, p. Asp299Gly, where Gly is the LOF allele (4,7)] in strong linkage disequilibrium with rs4986791: c.1196C>T, p.Thr399Ile, are proposed to act by attenuating the immune response against the immunogenic cell death caused by these agents (4–7). These associations were reflected in statistically significant differences in both progression-free and overall survival (OS) between patients bearing LOF and functional alleles in these genes when treated with these agents (hazard ratios [HRs] for LOF allele of 1.37–2.13; summarized in Supplementary Table 1, available online) (4–7,13,14). If validated, these variants could be used as biomarkers to target these toxic therapies to those most likely to benefit from them, resulting in less harm to patients and cost savings for health-care providers. Because anthracyclines and oxaliplatin are the mainstays of systemic treatment against two common cancers (breast and colorectal, respectively) (15,16) and because these LOF polymorphisms are relatively common (prevalence of 5% to 80% in populations of European descent), confirmation of this association could affect many thousands of patients each year in Europe and the United States alone. The purpose of this validation study was to confirm this association in the context of oxaliplatin treatment for CRC by analysis of two well-defined, prospectively treated cohorts from the Short Course in Oncology Therapy (SCOT) and COIN/COIN-B trials (17,18), encompassing both early-stage and advanced disease.

## Methods

### Clinical Trials

Details of the SCOT (ISRCTN59757862), COIN (ISRCTN27286448), and COIN-B (ISRCTN38375681) trials have been published previously (17–20). Briefly, the SCOT trial compared the efficacy of 12 weeks of oxaliplatin-based adjuvant chemotherapy with the previous standard of care of 24 weeks of treatment in high-risk, stage II (defined as one or more of: pT4 primary tumor, tumor obstruction, fewer than 10 lymph nodes harvested, grade 3 histology, perineural invasion, or extramural venous or lymphatic vascular invasion), or stage III colon or rectal cancer. The trial randomized 6088 patients between March 2008 and November 2013, of whom 6065 consented for their data to be used for the intention to treat analyses. At its primary analysis, the attenuated course of chemotherapy was confirmed to be noninferior to the standard of care (HR = 1.01, 95% CI = 0.91 to 1.11, test for noninferiority *P* = .012) (17). As part of the study, participants at selected centers were invited to participate in a translational substudy, the TransSCOT study. Tissue and blood samples were collected from these patients and constitutional DNA was extracted for translational studies. Following informed consent, 3109 patients provided samples for analysis. The COIN trial examined both the efficacy of the anti-EGFR monoclonal antibody cetuximab added to oxaliplatin-based chemotherapy and the impact of interrupting treatment in patients with stable or responding metastatic CRC after 12 to 16 weeks of systemic therapy (18). The trial recruited 2445 patients between March 2005 and May 2008. At its primary analysis, no statistically significant difference was observed between the chemotherapy-only and the chemotherapy plus cetuximab groups (20), and the comparison between intermittent and continuous chemotherapy failed to confirm noninferiority of interrupting treatment (18). The COIN-B study compared intermittent chemotherapy with either intermittent or continuous cetuximab in 226 patients with metastatic CRC (19). Among 169 patients with *KRAS* wild-type disease, analysis suggested greater activity of continuous cetuximab, though this difference was not statistically significant. As part of ancillary translational studies, 2244 study participants in COIN and COIN-B donated blood samples for DNA extraction and analysis. Given their similar patient populations and treatments (21), the COIN and COIN-B biomarker cohorts were combined for all analyses.

### DNA Extraction, Genotyping, and Imputation

DNA was extracted from EDTA-venous bloods using standard methods. After exclusion of samples that failed DNA extraction (n = 28) and those for which trial IDs were missing or duplicates (n = 14), 3067 DNA samples from the SCOT cohort were genotyped using the Global Screening Array (Illumina, San Diego, CA). Genotyping quality control entailed removal of any sample or single nucleotide polymorphism (SNP) with more than 2% missing data, any sample with an outlying heterozygosity rate, any sample with discordant reported sex and genotype imputed sex, and any SNP violating Hardy-Weinberg equilibrium at *P* less than 1 × 10^–10^ (n = 66 samples removed; n = 32 850 SNPs removed). Identity by descent analysis was conducted in PLINK 1.9 (22) and population stratification was examined using EIGENSTRAT (23). Related individuals (n = 8) were removed (IBD > 0.185) along with those with non-European ancestry (n = 54, as assessed by merging SCOT with HapMap release 23a and removing outliers based on eigenvector 1). Genotypes for 2939 remaining individuals were phased using SHAPEIT (24) and imputed using IMPUTE2 (25) and the UK10K + 1000 genomes merged reference panel. Of the SNPs analyzed in this study, rs3775291, rs4986790, and rs4986791 were directly genotyped. The fourth, rs867228, was imputed with an info score of 0.95. For this imputed SNP, genotype probabilities were converted to genotypes using gtool (http://www.well.ox.ac.uk/∼cfreeman/software/gwas/gtool.html) with a minimum probability threshold of .9 set for specifying per sample genotypes.

Cases from the COIN and COIN-B studies were genotyped using Affymetrix Axiom Arrays according to the manufacturer’s recommendations (Affymetrix, Santa Clara, CA) at the King Faisal Specialist Hospital and Research Center, Saudi Arabia (under IRB approval 2110033). We excluded individuals from analysis if they failed one or more of the following thresholds: overall successfully genotyped SNPs less than 95% (n = 122), discordant sex information (n = 8), classed as out of bounds by Affymetrix (n = 30), duplication or cryptic relatedness (identity by descent >0.185, n = 4), and evidence of non-white European ancestry by principal components analysis-based analysis in comparison with HapMap samples (n = 130). Imputation was performed using 1000 Genomes Project Pilot data as a reference panel (26). Genetic linkage of SNPs was determined by calculation of D’ and R2 using PLINK 1.9 (22).

### Statistical Analyses

Comparison between groups was made using unpaired Student *t* test for continuous variables (eg, age) and either χ^2^ or Fisher exact test for categorical variables (eg, mutation present vs absent, responder vs nonresponder). Biomarker analyses in this study were performed and are reported in accordance with the REMARK guidelines (27). All analyses were prespecified and are detailed in Supplementary Table 2 (available online). Survival endpoints included disease-free survival (DFS, defined as time from study randomization to CRC recurrence or death from any cause in SCOT only) and OS (defined as time from randomization to death from any cause in both cohorts). Progression-free survival was not used as an endpoint in the COIN/COIN-B trials in view of the difficulty in defining its duration in the context of intermittent chemotherapy, which was tested in both studies. Survival curves for SNP genotypes were plotted using the Kaplan-Meier method and analyzed by the log-rank test. Survival endpoints were also analyzed by univariate and multivariable Cox proportional hazards models, under additive, recessive, and dominant genetic models (eg, for rs867228, which has alleles A and C—of which C is the LOF allele—the additive model implies CC [2] vs CA [1] vs AA [0], modelled as a continuous variable; the recessive model implies CC [1] vs both CA and AA [0]; and the dominant model implies both CC and CA [1] vs AA). Proportionality of hazards was confirmed by inspection of scaled Schoenfeld residuals. For the multivariable analyses, adjustment was made for baseline demographic variables (age, sex), clinicopathological and molecular covariables of known prognostic value where available, and treatment type and schedule depending on the cohort. In the SCOT analyses, these comprised age, sex, disease site (colon vs rectum), primary tumor stage (pT1–2 vs pT3 vs pT4), nodal status (N0 vs N1 vs N2), treatment regimen (FOLFOX or CAPOX), and treatment duration (24 vs 12 weeks). In the COIN/COIN-B analyses, these comprised age, sex, disease site (colon vs rectum), World Health Organization (WHO) performance status (0 or 1 vs 2), primary tumor resection (unresected vs resected), tumor *KRAS*, *NRAS*, and *BRAF* mutation status (mutated vs wild type), patient white blood cell count (<10 000 cells per μL vs ≥10 000 cells per μL), cetuximab treatment (yes vs no), chemotherapy regimen (FOLFOX vs CAPOX), and chemotherapy schedule (intermittent vs continuous). In both cases, covariables were prespecified and no selection procedure (eg, backwards elimination) was performed. Models included all cases for which data were available and excluded those with missing data. *P* values for individual predictors in Cox models were calculated by the Wald test. Statistical analyses were performed in R version 3.4.4 (CRAN Corporation) and STATA version 13 (StataCorp, College Station, TX). All statistical tests were two-sided. Statistical significance was accepted at *P* less than .05. No correction for multiple testing was applied.

### Ethical Approval

Informed consent for the collection and analysis of samples was provided by study participants at the time of study recruitment under trial-specific ethical approval. Molecular analysis of samples from the SCOT cohort was performed under North West – Liverpool Central Research Committee approval (17/NW/0252). Molecular analysis of COIN/COIN-B samples was performed under REC approval (04/MRE06/60).

## Results

### Patient Characteristics and SNP Genotyping

**Figure 1.**
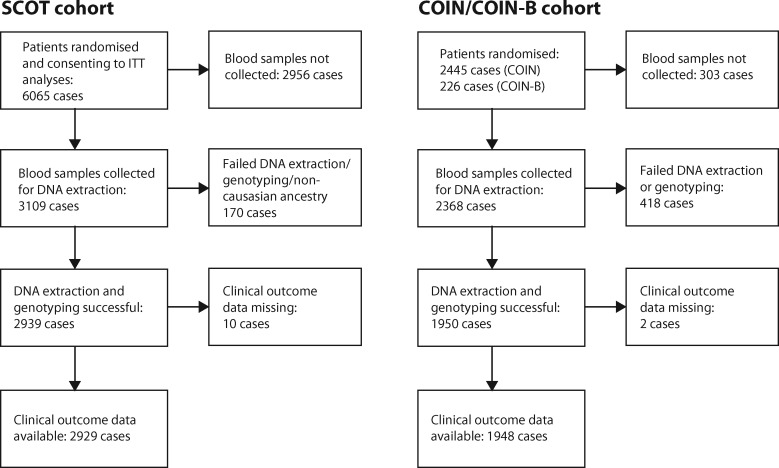
CONSORT diagram showing flow of patients analyzed in the study. ITT = intention to treat.

The CONSORT diagram demonstrating the flow of patients eligible for this biomarker study is shown in Figure 1. Demographic and clinicopathological characteristics of the 2929 SCOT cases with samples informative for this analysis were broadly similar to those of the SCOT trial population as a whole, although they differed statistically significantly, albeit modestly, from the nonbiomarker population in age, disease site, disease stage, and nodal status (Supplementary Table 2, available online). Characteristics of 2244 patients in the COIN/COIN-B biomarker subgroup were similar to the combined COIN/COIN-B trial population (not shown). Details of baseline demographic, clinicopathological, and molecular variables, and SNP genotypes in cases from both biomarker cohorts are provided in Table 1. Of 2929 patients in the SCOT cohort, 2728 (93.1%), 2924 (99.9%), and 2929 (100%) underwent successful genotyping or imputation and were informative for analysis of rs867228, rs3775291, and rs4986790/rs4986791 respectively. The slightly lower number of cases informative for rs867228 reflects the exclusion of those in which the genotype could not be imputed with high confidence. The corresponding numbers in the COIN/COIN-B cohort of 1948 patients were 1672 (85.6%), 1948 (100%), and 1948 (100%) respectively. The allelic frequencies of all SNPs in both cohorts were concordant with the reported population frequency in ExAC (28), EVS (29), and UK10K (30). As expected, rs4986790 and rs4986791 were in strong linkage disequilibrium in both the SCOT (D’ = 0.99 and *r*^2^^ ^= 0.93) and COIN/COIN-B (D’ = 0.99 and *r*^2^^ ^= 0.89) cohorts. Because analyses of these two SNPs individually yielded essentially identical results (Supplementary Figure 1, available online), we largely limited subsequent investigations to rs4986790.

**Table 1.** Baseline characteristics of SCOT and combined COIN/COIN-B cohorts

Variable	SCOT	COIN and COIN-B	*P*
	No. (%)	No. (%)	
Total	2929 (100)	1948 (100)	—
Median age, y (range)	65 (23–84)	53 (18–87)	<.001*
Sex			
Male	1795 (61.3)	1270 (65.2)	<.001†
Female	1134 (38.7)	678 (34.8)	
Unknown	0 (0.0)	0 (0.0)	
Disease stage			
II	585 (20.0)	0 (0.0)	—
III	2344 (80.0)	0 (0.0)	
IV	0 (0.0)	1948 (100.0)	
Unknown	0 (0.0)	0 (0.0)	
Primary tumor stage			
pT1	94 (3.2)	NA	—
pT2	285 (9.7)	NA	
pT3	1694 (57.8)	NA	
pT4	856 (29.2)	NA	
Unknown	0 (0.0)	NA	
Nodal stage			
N0	585 (20.0)	NA	—
N1	1695 (57.9)	NA	
N2	649 (22.2)	NA	
Unknown	0 (0.0)	NA	
Primary tumor location			
Colon	2346 (80.1)	1325 (67.9)	<.001†
Rectum	583 (19.9)	621 (31.9)	
Unknown	0 (0.0)	2 (0.2)	
Primary tumor resected			
No	0 (0.0)	821 (42.1)	—
Yes	2929 (100.0)	1127 (57.9)	
Unknown	0 (0.0)	0 (0.0)	
Peritoneal metastases			
No	NA	1519 (78.0)	—
Yes	NA	259 (13.3)	
Unknown	NA	170 (8.7)	
*KRAS* mutation status			
Wild-type	ND	989 (50.8)	—
Mutant	ND	636 (32.6)	
Unknown	ND	323 (16.6)	
*NRAS* mutation status			
Wild type	ND	1506 (77.3)	—
Mutant	ND	69 (3.6)	
Unknown	ND	373 (19.1)	
*BRAF* mutation status			
Wild type	ND	1438 (73.8)	—
Mutant	ND	143 (7.3)	
Unknown	ND	367 (18.9)	
*FPR1* rs867228 genotype			
AA	116 (4.0)	49 (2.5)	.003†
AC	813 (27.8)	444 (22.8)	
CC	1799 (61.4)	1179 (60.5)	
Unknown	201 (6.9)	276 (14.2)	
*TLR3* rs3775291 genotype			
CC	1486 (50.7)	934 (47.9)	.005†
CT	1207 (41.2)	810 (41.6)	
TT	231 (7.9)	204 (10.5)	
Unknown	5 (0.2)	0 (0.0)	
*TLR4* rs4986790 genotype			
AA	2581 (88.1)	1744 (89.5)	.11†
AG	333 (11.4)	200 (10.3)	
GG	15 (0.5)	4 (0.2)	
Unknown	0 (0.0)	0 (0.0)	
*TLR4* rs4986791 genotype			
CC	2568 (90.7)	1726 (88.6)	.12†
CT	344 (11.7)	218 (11.2)	
TT	17 (0.6)	4 (0.2)	
Unknown	0 (0.0)	0 (0.0)	

*Determined by two-sided unpaired Student *t* test. NA = not applicable; ND = not determined; pT = pathological tumor (T) stage; SCOT = Short Course in Oncology Therapy.

†Determined by two-sided χ^2^ test or Fisher exact test in the case of rs4986791 (in cases of SNP genotypes, values are calculated from cases in which SNP status was determined).

The effect sizes (hazard ratios) of each SNP detectable in multivariable analyses using recessive and dominant genetic models, based on a power (1-β) of 0.8 and a two-sided α of 0.05, are shown for both cohorts in Supplementary Table 4 (available online). For comparison with previous reports, our power to detect an association of identical effect size using the same (recessive) model to that previously reported for the *FPR1* rs867228 SNP was 1.0 and 0.995 for DFS and OS, respectively, in the SCOT cohort and 1.0 for OS in the COIN/COIN-B cohort. Our power to detect an association of the same effect size as that previously reported for the *TLR4* rs4986790 SNP using the same (dominant) model was 0.65 and 0.31 for DFS and OS, respectively, in the SCOT cohort and 0.96 for OS in the COIN/COIN-B cohort.

### Pattern Recognition SNPs and Clinical Outcome in the SCOT Cohort

**Figure 2.**
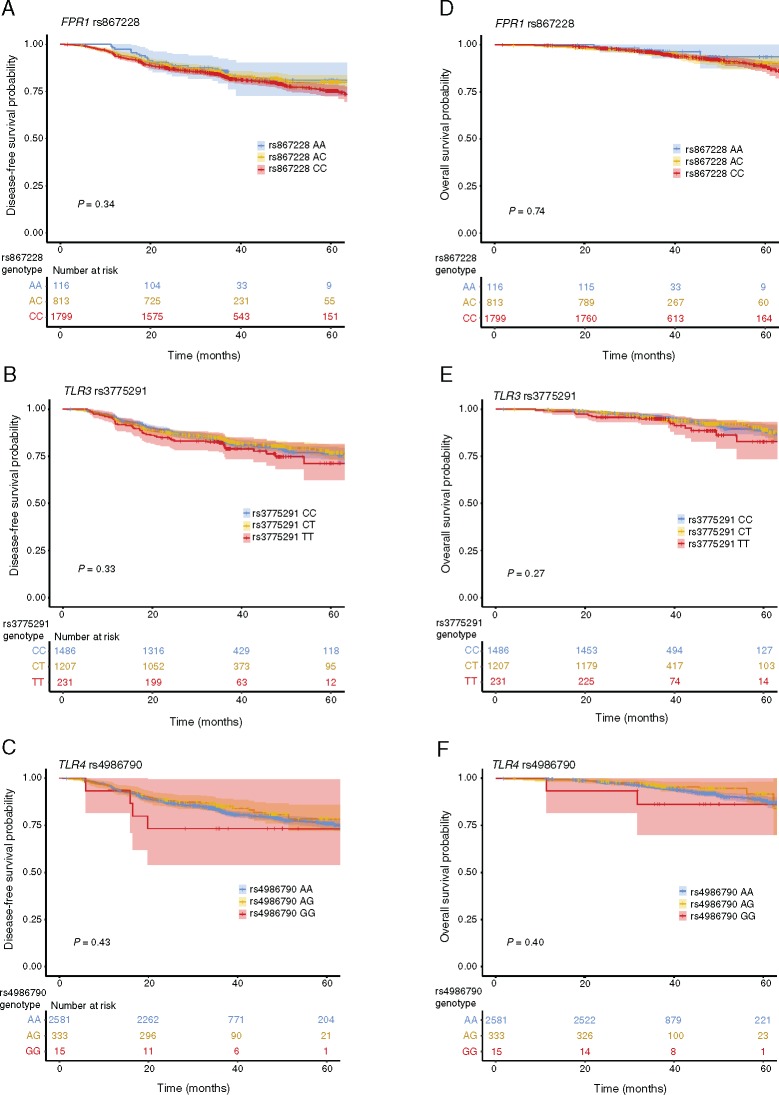
*FPR1*, *TLR3*, and *TLR4* loss of function (LOF) single nucleotide polymorphisms (SNPs), disease-free survival (DFS), and overall survival (OS) in Short Course in Oncology Therapy (SCOT) cohort. Kaplan Meier curves showing DFS for patients in SCOT cohort by pattern recognition receptor SNPs rs867228 (*FPR1* c.1037A>C p.Glu346Ala) (**A**), rs3775291 (*TLR3* c.1234C>T p.Leu412Phe) (**B**), and rs4986790 (*TLR4* c.896A>G, p.Asp299Gly) (**C**) (LOF allele/amino acid underscored in each case). Corresponding results for OS are shown in **D–F**. Analyses of the rs4987691 (*TLR4* c.1196C>T, p. Thr399Ile) polymorphism, which is strongly linked with rs4986790, were essentially identical to **C** and **F** and are provided as Supplementary Figure 1 (available online). Shaded areas represent 95% confidence intervals. *P* values indicate comparison of all groups by the two-sided log-rank test.

Biomarker analyses were performed with data used for the primary analysis of the SCOT trial, at which point the 2929 patients in the biomarker cohort had a median follow-up of 36.8 months, and 538 DFS events and 186 deaths had occurred (Table 2). Comparing survival curves by the log-rank test, univariate and multivariable Cox models demonstrated no statistically significant association of any SNP irrespective of genetic model imposed (Figure 2, Table 2, details of covariables in multivariable models provided in Supplementary Table 5, available online). This included models for which an association was previously reported for rs867228 (5) (recessive model, multivariable-adjusted HR for DFS = 1.19, 95% CI = 0.99 to 1.45, *P* = .07) and rs4986790 (4) (dominant model, multivariable-adjusted HR for DFS = 0.86, 95% CI = 0.65 to 1.13, *P* = .27) (Table 2, Supplementary Table 5, available online).

**Table 2.** Univariate and multivariable analyses of DFS and OS in SCOT cohort by LOF SNP*

Polymorphism/genetic model	No.	DFS events	OS events	Univariate analysis				Multivariable analysis				
				DFS		OS		DFS		OS		
				HR (95% CI)	*P*†	HR (95% CI)	*P*†	HR (95% CI)	*P*†	HR (95% CI)		*P*†
rs867228 (FPR1 c.1037A>C)	2728	487	167									
Additive	—	—	—	1.13 (0.96 to 1.32)	.15	1.09 (0.83 to 1.44)	.53	1.16 (0.98 to 1.37)	.08	1.10 (0.84 to 1.47)		.48
Recessive	—	—	—	1.15 (0.95 to 1.40)	.15	1.07 (0.77 to 1.49)	.67	1.19 (0.99 to 1.45)	.07	1.10 (0.79 to 1.53)		.56
Dominant	—	—	—	1.17 (0.73 to 1.88)	.50	1.40 (0.57 to 3.41)	.46	1.16 (0.73 to 1.87)	.53	1.32 (0.54 to 3.22)		.54
rs3775291 (TLR3 c.1234C>T)	2924	536	186									
Additive	—	—	—	1.05 (0.92 to 1.19)	.52	1.15 (0.93 to 1.44)	.29	1.02 (0.90 to 1.17)	.68	1.13 (0.91 to 1.41)		.27
Recessive	—	—	—	1.24 (0.92 to 1.66)	.15	1.46 (0.92 to 2.32)	.11	1.14 (0.85 to 1.52)	.38		1.32 (0.83 to 2.10)	.24
Dominant	—	—	—	1.01 (0.85 to 1.19)	.95	1.12 (0.84 to 1.49)	.44	1.00 (0.85 to 1.19)	.97	1.12 (0.84 to 1.49)		.44
rs4986790 (TLR4 c.896A>G)	2929	538	186									
Additive	—	—	—	0.92 (0.71 to 1.19)	.52	0.89 (0.57 to 1.39)	.62	0.89 (0.69 to 1.16)	.39	0.87 (0.56 to 1.36)		.54
Recessive	—	—	—	1.49 (0.55 to 4.00)	.42	1.93 (0.48 to 7.76)	.36	1.58 (0.59 to 4.25)	.36	1.82 (0.44 to 7.40)		.40
Dominant	—	—	—	0.89 (0.67 to 1.17)	.40	0.83 (0.51 to 1.35)	.45	0.86 (0.65 to 1.13)	.27	0.81 (0.50 to 1.32)		.39

*Both univariate and multivariable analyses use all informative cases. Hazard ratios show risk associated with reported LOF allele (underscored) for each SNP as follows: rs867228: *FPR1* c.1037A>C p.Glu346Ala; rs3775291: *TLR3* c.1234C>T, p.Leu412Phe; rs4986790: *TLR4* c.896A>G, p. Asp299Gly. Corresponding associations from rs4986791 (*TLR4* c.1196C>T, p.Thr399Ile), which is tightly linked to rs4986790, were essentially identical to those obtained from analysis of rs4986790 and are not shown. Multivariable-adjusted HRs were adjusted for age, sex, disease site (colon vs rectum), primary tumor stage (pT1–2 vs pT3 vs pT4), nodal status (N0 vs N1 vs N2), treatment regimen (FOLFOX or CAPOX), and treatment duration (24 vs 12 weeks). Prognostic associations of covariables are shown in Supplementary Table 5 (available online). CI = confidence interval; DFS = disease-free survival; HR = hazard ratio; LOF = loss of function; OS = overall survival; pT = pathological tumor (T) stage; SCOT = Short Course in Oncology Therapy.

†*P* values were calculated by two-sided Wald test.

A previous study reported that the association of the *FPR1* LOF polymorphism rs867228 was only evident in patients with functional *TLR3* or *TLR4*, consistent with their participation in the same pathway (5). We therefore examined this in the SCOT biomarker cohort after stratifying by *TLR3* (rs3775291) and *TLR4* (rs4986790) status. These analyses did not confirm the previously reported, statistically significant association with DFS in the context of either functional *TLR3* background (multivariable-adjusted HR for additive model = 1.02, 95% CI = 0.82 to 1.27, *P* = .85; recessive model HR = 1.01, 95% CI = 0.78 to 1.31, *P* = .91; dominant model HR = 1.09, 95% CI = 0.59 to 2.00, *P* = .78) or functional *TLR4* background (additive model HR = 1.17, 95% CI = 0.99 to 1.40, *P* = .07; recessive model HR = 1.20, 95% CI = 0.98 to 1.48, *P* = .08; dominant model HR = 1.31, 95% CI = 0.79 to 1.20, *P* = .30). Similarly, no statistically significant association of rs867228 with DFS was observed in cases with functional polymorphisms at both of these loci (multivariable-adjusted HR for additive model = 0.97, 95% CI = 0.77 to 1.21, *P* = .76; recessive model HR = 0.92, 95% CI = 0.70 to 1.22, *P* = .58; dominant model HR = 1.14, 95% CI = 0.60 to 2.16, *P* = .68) (Supplementary Figure 2, available online).

### Pattern Recognition SNPs, Clinical Outcome, and Oxaliplatin Response in the COIN/COIN-B Cohort

**Figure 3.**
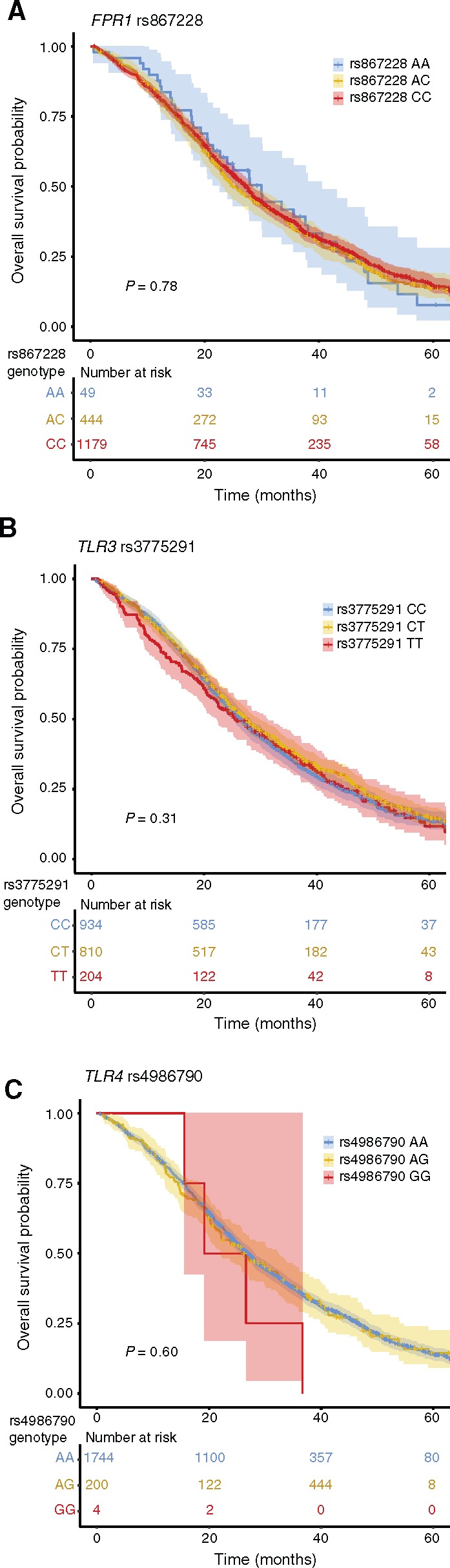
*FPR1*, *TLR3*, and *TLR4* loss of function (LOF) single nucleotide polymorphisms (SNPs) and overall survival (OS) in COIN/COIN-B cohort. Kaplan Meier curves showing OS for patients in combined COIN/COIN-B cohort by pattern recognition receptor SNPs rs867228 (*FPR1* c.1037A>C p.Glu346Ala) (**A**), rs3775291 (*TLR3* c.1234C>T p.Leu412Phe) (**B**), and rs4986790 (*TLR4* c.896A>G, p.Asp299Gly) (**C**) (LOF allele/amino acid underscored in each case). Analyses of the rs4987691 (*TLR4* c.1196C>T, p. Thr399Ile) polymorphism, which is strongly linked with rs4986790, were essentially identical to **C** and are not shown. Shaded areas represent 95% confidence intervals. *P* values indicate comparison of all groups by the two-sided log-rank test.

Corresponding analyses were performed on the COIN/COIN-B cohort in which the median follow-up of the 1948 patients was 23.2 months, by which time 1453 deaths had occurred. Similar to the SCOT analyses, there was no statistically significant association of either SNP with OS by either log-rank test or univariate or multivariable Cox regression, regardless of model (Figure 3 , Table 3, details of covariables in multivariable models provided in Supplementary Table 6, available online). Again, this included the recessive model for rs867228 (5) (multivariable-adjusted HR for OS = 0.92, 95%CI = 0.63 to 1.34, *P* = .66), and the dominant model for rs4986790 (4) (multivariable-adjusted HR for OS = 1.08, 95% CI = 0.90 to 1.31, *P* = .40) (Table 3, Supplementary Table 6, available online). Likewise, prespecified subgroup analyses stratified by *TLR3* and *TLR4* status revealed no evidence of an association between *FPR1* status and OS in the context of functional *TLR3* (multivariable-adjusted HR for additive model = 0.93, 95% CI = 0.78 to 1.10, *P* = .37; recessive model HR = 0.91, 95% CI = 0.56 to 1.48, *P* = .71; dominant model HR = 0.91, 95% CI = 0.74 to 1.12, *P* = .36), or functional *TLR4* (additive model HR = 1.03, 95% CI = 0.90 to 1.17, *P* = .66; recessive model HR = 1.00, 95% CI = 0.68 to 1.49, *P* = .99; dominant model HR = 1.04, 95% CI = 0.89 to 1.21, *P* = .62). Similar to the results from the SCOT cohort, no statistically significant association was observed in cases with functional polymorphisms at both loci (multivariable-adjusted HR for additive model = 0.99, 95% CI = 0.82 to 1.19, *P* = .87; recessive model = 1.22, 95% CI = 0.73 to 2.04, *P* = .43; dominant model HR = 0.95, 95% CI = 0.76 to 1.18, *P* = .63) (Supplementary Figure 3, available online).

**Table 3.** Univariate and multivariable analyses of OS in combined COIN/COIN-B cohort by LOF SNP*

Polymorphism/genetic model	Univariate analysis				Multivariable analysis			
	No.	OS events	HR (95% CI)	*P*†	No.	OS events	HR (95% CI)	*P*†
rs867228 (FPR1 c.1037A>C)	1672	1241	—	—	1336	970	—	—
Additive	—	—	1.03 (0.93 to 1.14)	.60	—	—	0.99 (0.88 to 1.12)	.91
Recessive	—	—	0.98 (0.71 to 1.37)	.93	—	—	0.92 (0.63 to 1.34)	.66
Dominant	—	—	1.04 (0.92 to 1.18)	.52	—	—	1.05 (0.90 to 1.23)	.53
rs3775291 (TLR3 c.1234C>T)	1948	1453	—	—	1563	1150	—	—
Additive	—	—	0.98 (0.91 to 1.06)	.67	—	—	0.97 (0.89 to 1.06)	.56
Recessive	—	—	1.07 (0.90 to 1.26)	.45	—	—	1.08 (0.90 to 1.31)	.41
Dominant	—	—	0.94 (0.86 to 1.05)	.31	—	—	0.93 (0.83 to 1.04)	.20
rs4986790 (TLR4 c.896A>G)	1948	1453	—	—	1563	1150	—	—
Additive	—	—	1.03 (0.88 to 1.21)	.71	—	—	1.10 (0.91 to 1.33)	.31
Recessive	—	—	1.65 (0.61 to 4.40)	.31	—	—	2.91 (0.93 to 9.12)	.07
Dominant	—	—	1.02 (0.86 to 1.20)	.81	—	—	1.08 (0.90 to 1.31)	.40

*Both univariate and multivariable analyses use all informative cases (ie, cases lacking covariable data were excluded from multivariable models). Hazard ratios show risk associated with reported LOF allele (underscored) for each SNP as follows: rs867228: *FPR1* c.1037A>C p.Glu346Ala; rs3775291: *TLR3* c.1234C>T, p.Leu412Phe; rs4986790: *TLR4* c.896A>G, p. Asp299Gly. Corresponding associations from rs4986791 (*TLR4* c.1196C>T, p.Thr399Ile), which is tightly linked to rs4986790, were essentially identical to those obtained from analysis of rs4986790 and are not shown. Multivariable-adjusted HRs are adjusted for age, sex, disease site (colon vs rectum), World Health Organization (WHO) performance status (0 or 1 vs 2), primary tumor resection (unresected vs resected), tumor *KRAS*, *NRAS*, and *BRAF* mutation status (mutated vs wild type), patient white blood cell count (<10 000 cells/μL vs ≥10 000 cells/μL), cetuximab treatment (yes vs no), chemotherapy regimen (FOLFOX vs CAPOX), and chemotherapy schedule (intermittent vs continuous). Prognostic associations of covariables are shown in Supplementary Table 6 (available online). CI = confidence interval; HR = hazard ratio; LOS = loss of function; OS = overall survival; pT = pathological tumor (T) stage; SNP = single nucleotide polymorphism.

†*P* values were calculated by two-sided Wald test.

An additional analysis according to radiological response to oxaliplatin-based chemotherapy after 12 weeks of therapy [complete or partial response vs stable or progressive disease by RECIST 1.0 (31)] revealed no difference in the proportions of functional and LOF alleles between responders and nonresponders for rs867228 (*P* = .90, χ^2^ test), rs3775291 (*P* = .68, χ^2^ test), or rs4986790 (*P* = .64, Fisher exact test).

## Discussion

Previous studies have suggested that LOF polymorphisms in the pattern recognition receptors *FPR1* (rs867228), *TLR3* (rs3775291), and *TLR4* (rs4986790/rs4986791) decrease the presentation of ligand to the innate immune system by dying cells (3–7). This, in turn, is proposed to reduce the efficacy of anthracycline and oxaliplatin chemotherapy, the activities of which depend in part on the induction of immunogenic cell death (3–5,7). In this study of nearly 5000 patients with CRC treated with oxaliplatin, we failed to confirm any of these associations. The 95% confidence intervals for the association of each SNP with DFS and OS in the SCOT cohort and OS in the COIN/COIN-B cohort all included the estimate of no effect. Although our data by no means exclude an immunomodulatory effect of these SNPs, they suggest that they are very unlikely to be clinically useful as predictive biomarkers for oxaliplatin benefit in CRC. The discordance between our results and those from previous studies may be explained by the increased risk of false-positive associations in the smaller cohorts they used, and in the case of rs867228, an apparent misclassification of the functional and LOF alleles in the survival analyses (the functional *FPR1* allele c.1037A, p.346Glu appeared to be incorrectly classified as LOF in all analyses in the study by Vacchelli et al.) (5). Our results underscore the importance of validation of encouraging findings from modestly sized studies in large, meticulously curated trial cohorts, even where preclinical data provide a plausible mechanism for an association.

Strengths of our study include its large size, defined clinical trial cohorts, standardized therapy, comprehensively annotated clinicopathological variables, and, in the case of the COIN/COIN-B cohort, molecular variables and mature outcome data. Consequently, our analyses were powered to detect even a modest association of most SNPs with clinical outcome and had a power of greater than 0.95 to detect an association of similar strength to that previously reported for the rs867228 and rs4986790 LOF variants (4,6). Limitations include the lack of molecular profiling in the SCOT trial, which meant that we were unable to test for an association of the SNPs with clinical outcome in specific tumor subgroups such as those with enhanced immunogenicity due to defective DNA mismatch repair or *POLE* exonuclease domain mutation.

In summary, in this study of two large clinical trial cohorts, we find no evidence that LOF SNPs in the pattern recognition receptors *FPR1*, *TLR3,* and *TLR4* are associated with differential benefit from oxaliplatin in CRC. Future studies may better define the complex relationship between cytotoxic therapeutic-induced cell death, pattern recognition SNPs, and the innate immune system.

## Funding

This work was supported by The Oxford NIHR Comprehensive Biomedical Research Centre, Cancer Research UK (C6199/A10417 and C399/A2291), the European Union Seventh Framework Programme (FP7/207– 2013) grant 258236 collaborative project SYSCOL, European Research Council project EVOCAN, and core funding to the Wellcome Trust Centre for Human Genetics from the Wellcome Trust (090532/Z/09/Z). The SCOT trial was supported by the Medical Research Council (transferred to NETSCC—Efficacy and Mechanism Evaluation; grant reference G0601705), the Swedish Cancer Society, and Cancer Research UK Core Clinical Trials Unit Funding (funding reference C6716/A9894). The TRIAL sponsor was NHS Greater Glasgow & Clyde and University of Glasgow (Eudract reference 2007–003957–10; ISRCTN number 23516549). COIN and COIN-B were coordinated by the Medical Research Council Clinical Trials Unit and conducted with the support of the National Institutes of Health Research Cancer Research Network. COIN and COIN-B translational studies were supported by the Bobby Moore Fund from Cancer Research UK, Tenovus, the Kidani Trust, Cancer Research Wales, and the National Institute for Social Care and Health Research Cancer Genetics Biomedical Research Unit.

SB is funded by an MRC Clinical Research Training Fellowship. CP is funded by a University of Birmingham Fellowship. NAA, BFM, and SMW were funded and supported by KFSHRC. RSH is supported by Cancer Research UK. DNC is funded by a Health Foundation/Academy of Medical Sciences Clinician Scientist Fellowship.

The cost of open access publication was provided by core funding to the Wellcome Centre for Human Genetics from the Wellcome Trust (203141/Z/16/Z).

## Notes

Division of Cancer and Genetics, School of Medicine, Cardiff University, Cardiff, UK (VG, MGS, JPC); Wellcome Centre for Human Genetics, University of Oxford, Oxford, UK (SB, EJ, LG, DNC); Cancer Genetics and Evolution Laboratory, Institute of Cancer and Genomic Sciences, University of Birmingham, Edgbaston, Birmingham, UK (SB, EJ, IT); Gastrointestinal Cancer Genetics Laboratory, Institute of Cancer and Genomic Sciences, University of Birmingham, Edgbaston, Birmingham, UK (CP); Southampton University Hospital NHS Foundation Trust, Southampton, UK (TI, TSM); Department of Oncology, Old Road Campus Research Building, University of Oxford, Oxford, UK (RK, EJ, HW); Christie Hospital NHS Foundation Trust, Manchester, UK (MPS); Cancer Research UK Clinical Trials Unit, Institute of Cancer Sciences, University of Glasgow, Glasgow, UK (JP, AH, JM); MRC Clinical Trials Unit at UCL, London, UK (RK); Institute of Psychological Medicine and Clinical Neurosciences, School of Medicine, Cardiff University, Cardiff, UK (VE-P); Department of Genetics, King Faisal Specialist Hospital and Research Center, Riyadh, Saudi Arabia (NAA-T, BFM, SMW); Division of Genetics and Epidemiology, The Institute of Cancer Research, London, UK (RSH); NIHR Oxford Comprehensive Biomedical Research Centre, Oxford University Hospitals NHS Foundation Trust, Oxford, UK (DNC).

The authors have no disclosures. The funders had no role in the design of the study; the collection, analysis, and interpretation of the data; the writing of the manuscript; and the decision to submit the manuscript for publication. The views expressed are those of the authors and not necessarily those of the NHS, the NIHR, the Department of Health, or the Wellcome Trust. We are grateful to the participants in the SCOT, COIN, and COIN-B trials who consented for the donation of samples for research, and to the investigators and pathologists who recruited patients and collected samples.

Author contributions: Study design: CP, JPC, IT, DNC; data collection: SB, CP, EJ, TI, RK, MPS, JP, AH, JM, MGS, EJ, HW, LG, TSM, RK, VE-P, NA-T, BFM, SMW, RSH; data analysis: VG, CP, JPC, IT, DNC; manuscript writing: DNC; manuscript approval: all authors.

## Supplementary Material

djy215_Supplementary_Data
