# Supplementary material for: Pattern Recognition Receptor Polymorphisms as Predictors of Oxaliplatin Benefit in Colorectal Cancer
Source: J Natl Cancer Inst. 2019 Jan 14;111(8):828–36. doi: 10.1093/jnci/djy215 (PMC6695319; doi:10.1093/jnci/djy215)
Supplement: djy215_Supplementary_Data [file djy215_supplementary_data.pdf]

Supplementary material to Gray et al.

Pattern recognition receptor polymorphisms as predictors of oxaliplatin benefit in colorectal cancer

Supplementary Table 1. Results of previous analyses of pattern recognition receptor loss of function polymorphisms and clinical outcome in patients treated with anthracyclines or oxaliplatin

SNP	Loss of function variant (underlined)	Disease type	Treatment	Sample size	Genetic model for association	Endpoint	Association of loss of function allele with clinical outcome		Ref.
							HR (95% CI)	*P*	
rs867228	*FPR1*: c.1037A>C, p.Glu346Ala	Early-stage breast cancer	Anthracycline-based chemotherapy	731	Dominant	Metastasis-free survival	1.41 (1.06–2.08)	0.02*	Vacchelli et al. 2015 [1]
						Overall survival	1.58, (1.19–2.08)	0.002*	
		Metastatic colorectal cancer	Oxaliplatin-based chemotherapy	311	Recessive	Progression-free survival	1.85 (1.12–3.13)	0.02	Vacchelli et al. 2015 [1]
						Overall survival	2.13 (1.28–3.57)	0.004	
rs3775291	*TLR3*: c.1234C>T, p.Leu412Phe,	Breast cancer	Various	715	Recessive	Recurrence-free survival	3.53 (1.98–6.31)	<0.01	Chen et al. 2015 [2]
		Colorectal cancer (all stages)	Various	565	Recessive	CRC-specific survival	1.93 (1.14-3.28)	0.03	Castro et al. 2011 [3]
rs4986790†	TLR4 c.896A>G, p. Asp299Gly	Node positive breast cancer	Anthracycline-based chemotherapy	280	Dominant	Metastasis-free survival	–‡	0.03	Apetoh et al. 2007 [4]
		Metastatic colorectal cancer	Oxaliplatin-based chemotherapy	338	Dominant	Progression-free survival	1.37 (1.0–1.88)	0.05	Tesniere et al. 2010 [5]
						Overall survival	1.38 (1.0–1.92)	0.05	

* result for loss of function allele (LOF) as reported in original publication, with apparent misclassification of functional and LOF alleles (the functional *FPR1* allele c.1037A, p.346Glu was incorrectly classified as LOF in all analyses). † rs4986790 is in strong linkage disequilibrium with rs4986791: *TLR4* c.1196C>T, p.Thr399Ile; the two have been analysed in combination in previous studies. HR = hazard ratio, 95% CI = 95% confidence interval. ‡ Worse with LOF allele. A two-sided log rank test was used to calculate the *P* value

Supplementary Table 2. Biomarker analyses performed and reported in this study*

Analysis	Objective	Endpoints	Population	Methods	Reported
Association of candidate constitutional loss of function SNPs in *FPR*1, *TLR3* and *TLR4* with clinical outcome	Primary	DFS, OS	All informative cases from SCOT cohort	Kaplan-Meier plots, log-rank test, univariable and multivariable Cox proportional hazards models using additive, recessive and dominant models	Main text, Table 2, Figure 2
Association of constitutional loss of function SNPs in *FPR1*, *TLR3* and *TLR4* with clinical outcome	Primary	OS	All informative cases from COIN/COIN-B cohort	Kaplan-Meier plots, log-rank test, univariable and multivariable Cox proportional hazards models using additive, recessive and dominant models	Main text, Table 3, Figure 3
Association of constitutional loss of function SNPs in *FPR1* with clinical outcome stratified by *TLR3* and *TLR4* status	Exploratory	DFS	Informative cases from SCOT cohort stratified by *TLR3* and *TLR4* status	Multivariable-adjusted Cox proportional hazards models	Main text, Figure S2,
Association of constitutional loss of function SNPs in *FPR1* with clinical outcome stratified by *TLR3* and *TLR4* functional status	Exploratory	OS	Informative cases from COIN cohort stratified by *TLR3* and *TLR4* status	Multivariable-adjusted Cox proportional hazards models	Main text, Figure S3,

* DFS– disease-free survival; OS – overall survival; HR – hazard ratio

Supplementary Table 3. Comparison of SCOT trial cases used in this biomarker study with those not informative for biomarker analysis and the total SCOT trial population

Variable	Cases used in this study		Non-informative for biomarker analysis		SCOT trial intention to treat population		*P* (biomarker vs. non-biomarker)
	No.	%	No.	%	No.	%	
Total	2929	48.3	3136	51.7	6065	100	
Age (years)							
Median (range)	65 (23–84)		64 (20-85)		65 (20–85)		7.8×10^{-3} †
Sex							
Male	1795	61.3	1877	59.9	3672	60.5	0.27 ‡
Female	1134	38.7	1259	40.1	2393	39.5	
Unknown	0	0.0	0	0.0	0	0.0	
Disease stage							
II	585	20.0	529	16.9	1114	18.4	0.002 ‡
III	2344	80.0	2607	83.1	4951	81.6	
Unknown	0	0.0	0	0.0	0	0.0	
Primary tumor stage							
pT1	94	3.2	97	3.1	191	3.1	0.39 ‡
pT2	285	9.7	280	8.9	565	9.3	
pT3	1694	57.8	1785	56.9	3479	57.4	
pT4	856	29.2	974	31.1	1830	30.2	
Unknown	0	0.0	0	0.0	0	0.0	
Nodal stage							
N0	585	20.0	529	16.9	1114	18.4	<0.001 ‡
N1	1695	57.9	1752	55.9	3447	56.8	
N2	649	22.2	855	27.3	1504	24.8	
Unknown	0	0.0	0	0.0	0	0.0	
Primary tumor location							
Colon	2346	80.1	2621	83.6	4967	81.9	<0.001 ‡
Rectum	583	19.9	515	16.4	1098	18.1	
Unknown	0	0.0	0	0.0	0	0.0	

pT –pathological tumor (T) stage. † determined by two-sided unpaired Student's t-test. ‡ determined by two-sided Chi square test

Supplementary Table 4. Study power and effect sizes detectable in multivariable analyses*

SNP/ Genetic model	SCOT cohort					COIN/COIN-B cohort		
	No. cases	Disease-free survival		Overall survival		No. cases	Overall survival	
		No. events	HR detectable	No. events	HR detectable		No. events	HR detectable
rs867228 (*FPR1* c.1037A>C)	2728	487	–	167		1336	970	
Recessive	–		1.32		1.59			1.22
Dominant	–		1.89		2.94			1.37
rs3775291 (*TLR3* c.1234C>T)	2924	536		186		1563	1150	
Recessive	–		1.56		2.13			1.35
Dominant	–		1.28		1.52			1.18
rs4986790 (*TLR4* c.896A>G)	2929	538		186		1563	1150	
Recessive	–		5.88		20.0			3.23
Dominant	–		1.45		1.89			1.35

* Calculation of hazard ratio (HR) detectable for each SNP and model are based on size, failure probability and SNP prevalence in each cohort and are based on a power (1-β) of 0.8 and a two-sided α of 0.05.

Supplementary Table 5. Prognostic associations of factors included in multivariable analysis of SCOT cohort*

Variable	No.	%	Multivariable analysis			
			Disease-free survival		Overall survival	
			HR (95% CI)	*P*†	HR (95% CI)	*P*†
Age (continuous)	–	–	1.00 (0.99–1.01)	0.66	1.02 (1.01–1.04)	0.006
Sex						
Male	1795	61.3	1.0 (ref)	–	1.0 (ref)	–
Female	1134	38.7	0.85 (0.71–1.02)	0.08	1.03 (0.77–1.39)	0.82
Disease site						
Colon	2346	80.1	1.0 (ref)	–	1.0 (ref)	–
Rectum	583	19.9	1.08 (0.86–1.36)	0.49	0.92 (0.60–1.40)	0.68
Primary tumour stage						
pT1-2	379	12.9	1.0 (ref)	–	1.0 (ref)	–
pT3	1694	57.8	2.30 (1.57–3.36)	<0.001	1.94 (0.97–3.90)	0.060
pT4	856	29.2	3.92 (2.64–5.81)	<0.001	4.46 (2.21–9.03)	<0.001
Nodal stage						
N0	585	20.0	1.0 (ref)	–	1.0 (ref)	–
N1	1695	57.9	1.97 (1.51–2.57)	<0.001	2.10 (1.34–3.27)	<0.001
N2	649	22.2	3.74 (2.84–4.91)	<0.001	4.30 (2.75–6.72)	0.002
Chemotherapy regimen						
CAPOX	937	32.0	1.0 (ref)	–	1.0 (ref)	–
FOLFOX	1992	68.0	0.99 (0.83–1.19)	0.94	1.08 (0.79–1.47)	0.62
Chemotherapy duration						
12 weeks	1461	(9.9	1.0 (ref)	–	1.0 (ref)	–
24 weeks	1468	50.1	0.94 (0.79–1.19)	0.45	0.99 (0.74–1.32)	0.95

*Analyses use all informative cases from total biomarker study population of 2929 cases, in which 538 DFS events and 186 deaths occurred during follow-up. Hazard ratios are adjusted for all other covariables listed. DFS – disease-free survival; OS – overall survival; HR – hazard ratio; 95% CI – 95% confidence interval; pT – pathological tumor (T) stage. † *P* values calculated by two-sided Wald test.

Supplementary Table 6. Prognostic associations of factors included in multivariable analysis of combined COIN/COIN-B cohort*

Variable	No.	%	Overall survival	
			**HR (95% CI)**	***P*†**
Age (continuous)	–	–	1.01 (1.00 – 1.01)	0.10
Sex				
Male	1039	66.5	1.0 (ref)	
Female	524	33.5	1.08 (0.95 – 1.22)	0.23
Disease site				
Colon	1064	68.1	1.0 (ref)	
Rectum	499	31.9	0.86 (0.76 – 0.98)	0.02
WHO performance status				
0-1	1450	92.8	1.0 (ref)	
2	113	7.2	1.50 (1.20 – 1.87)	<0.001
Primary tumor resected				
No	606	38.8	1.0 (ref)	
Yes	957	61.2	0.74 (0.65 – 0.83)	<0.001
White cell count				
<10,000/mcL	1117	71.5	1.0 (ref)	
≥10,000/mcL	446	28.5	1.64 (1.43 – 1.87)	<0.001
KRAS mutation status				
Wild-type	969	62.0	1.0 (ref)	
Mutant	594	38.0	1.51 (1.33 – 1.72)	<0.001
NRAS mutation status				
Wild-type	1494	95.6	1.0 (ref)	
Mutant	69	4.4	1.55 (1.17 – 2.06)	0.002
BRAF mutation status				
Wild-type	1422	91.0	1.0 (ref)	
Mutant	141	9.0	2.43 (1.98 – 2.97)	<0.001
Cetuximab treatment				
No	943	60.3	1.0 (ref)	
Yes	620	39.7	1.05 (0.92 – 1.20)	0.45
Chemotherapy regimen				
CAPOX	947	60.6	1.0 (ref)	
FOLFOX	616	39.4	0.95 (0.84 – 1.07)	0.41
Chemotherapy arm				
Continuous	970	62.1	1.0 (ref)	
Intermittent	593	37.9	1.07 (0.94 – 1.22)	0.31

* Analyses use all informative cases from total biomarker study population of 1563 cases, in which 1150 deaths occurred during follow-up. Hazard ratios are adjusted for all other covariables listed. HR – hazard ratio; 95% CI – 95% confidence interval; WHO – World Health Organisation; pT – pathological tumor (T) stage; CAPOX – capecitabine and oxaliplatin; FOLFOX –infusional 5-fluorouracil and oxaliplatin. † *P* values calculated by two-sided Wald test.

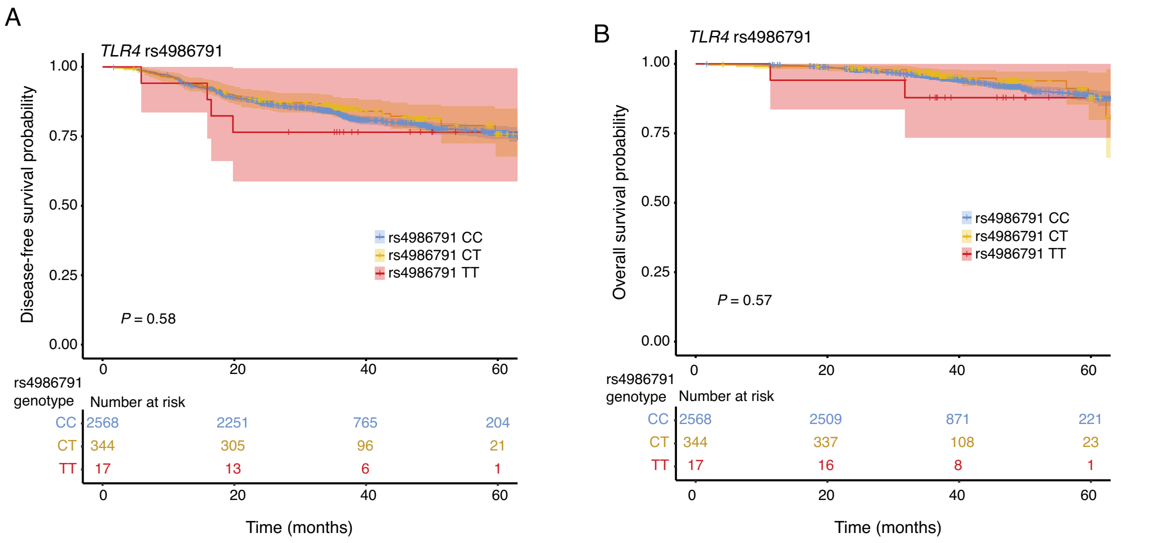

Supplementary Figure 1. Association of rs4986791 with disease-free survival in SCOT cohort

Kaplan Meier curves showing disease-free survival for patients in SCOT cohort by rs4986791 (*TLR4* c.1196C>T, p.Thr399Ile) genotype. Shaded areas represent 95% confidence intervals (95% CIs). *P* values indicate comparison of all groups by two-sided log-rank test.

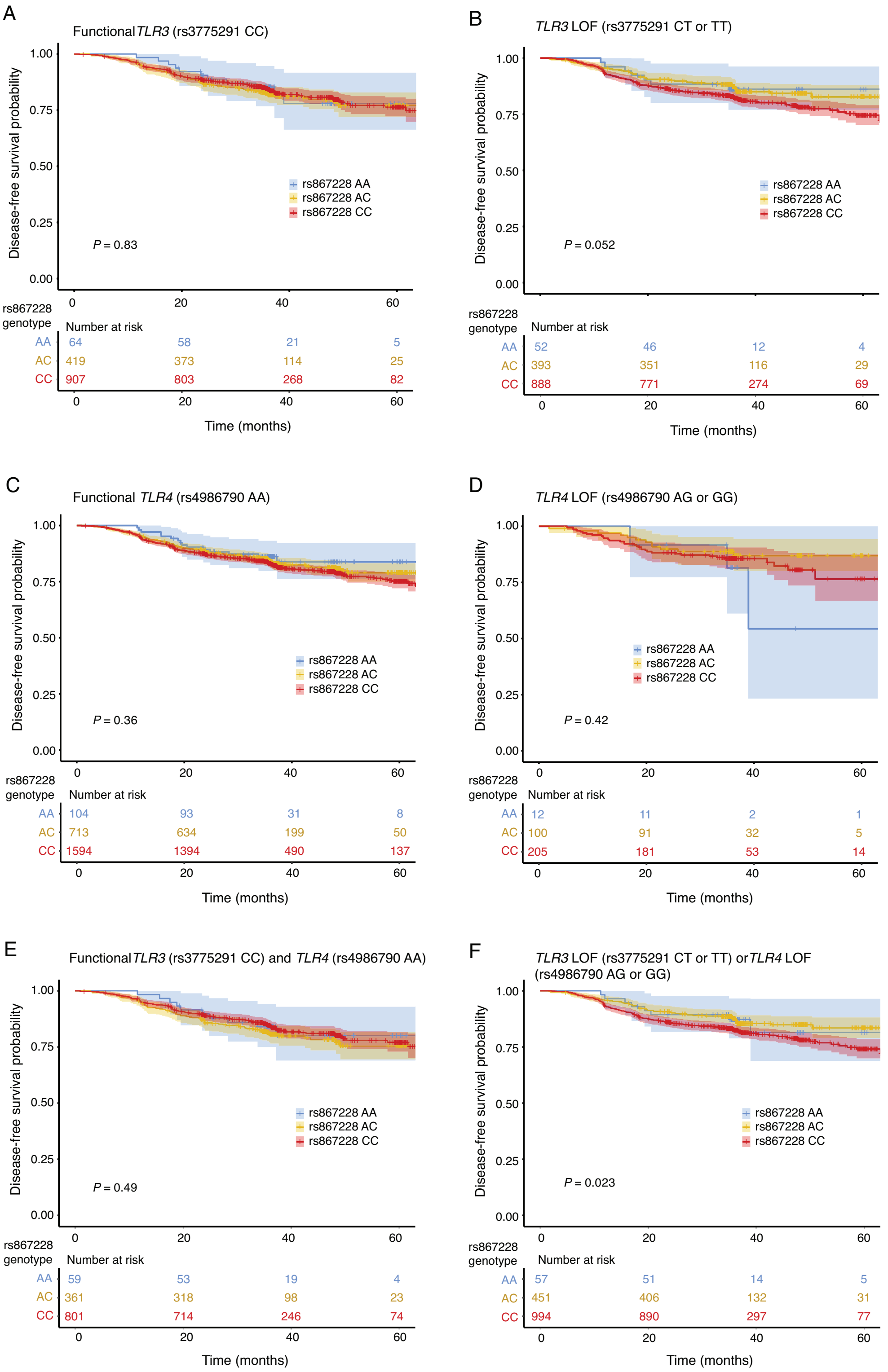

Supplementary Figure 2. Association of *FPR1* rs867228 with disease-free survival according to *TLR3* and *TLR4* functional status in SCOT cohort

Kaplan Meier curves showing disease-free survival for patients in SCOT cohort by *FPR1* rs867228 genotype (*FPR1* c.1037A>C p.Glu346Ala), *TLR3* and *TLR4* status. Upper panels show associations in cases homozygous for the functional rs3775291reference allele (*TLR3* c.1234C, p. Leu412) (A), and in cases heterozygous or homozygous for the rs3775291 loss of function (LOF) polymorphism (*TLR3* c.1234C>T p.Leu412Phe) (B). Middle panels show associations of rs867228 genotype in cases homozygous for the functional rs4986790 reference allele (*TLR4* c.896A, p. Asp299) cases (C), and in cases heterozygous and homozygous for the rs4986790 LOF polymorphism (*TLR4* c.896A>G, p. Asp299Gly) (D). Corresponding analyses for the rs4987691 (*TLR4* c.1196C>T, p. Thr399Ile) polymorphism, which is strongly linked with rs4986790, were essentially identical and are not shown. Lower panels show associations of rs867228 genotype in cases with functional alleles at both *TLR3* and *TLR4* (E) and in cases with LOF polymorphisms at either locus (F). Shaded areas represent 95% confidence intervals (95% CIs). *P* values indicate comparison of all groups by two-sided log-rank test.

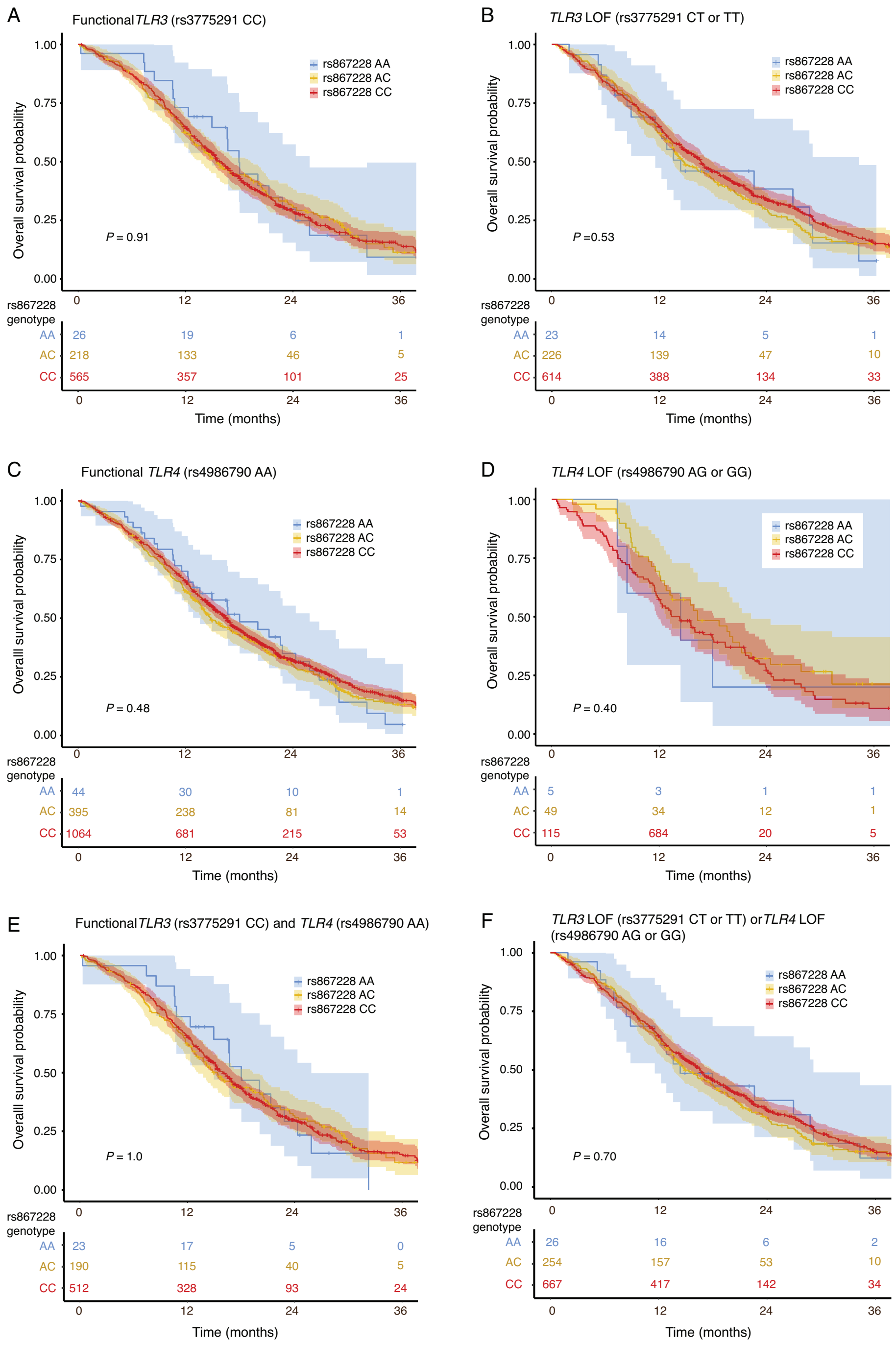

Supplementary Figure 3. Association of *FPR1* rs867228 with overall survival according to *TLR3* and *TLR4* functional status in COIN/COIN-B cohort

Kaplan Meier curves showing disease-free survival for patients in COIN/COIN-B cohort by *FPR1* rs867228 genotype (*FPR1* c.1037A>C p.Glu346Ala), *TLR3* and *TLR4* status. Upper panels show associations in cases homozygous for the functional rs3775291reference allele (*TLR3* c.1234C, p. Leu412) (A), and in cases heterozygous or homozygous for the rs3775291 loss of function (LOF) polymorphism (*TLR3* c.1234C>T p.Leu412Phe) (B). Middle panels show associations of rs867228 genotype in cases homozygous for the functional rs4986790 reference allele wild-type (*TLR4* c.896A, p. Asp299) cases (C), and in cases heterozygous and homozygous for the rs4986790 LOF polymorphism (*TLR4* c.896A>G, p. Asp299Gly) (D). Corresponding analyses for the rs4987691 (*TLR4* c.1196C>T, p. Thr399Ile) polymorphism, which is strongly linked with rs4986790, were essentially identical and are not shown. Lower panels show associations of rs867228 genotype in cases with functional alleles at both *TLR3* and *TLR4* (E) and in cases with LOF polymorphisms at either locus (F). Shaded areas represent 95% confidence intervals (95% CIs). *P* values indicate comparison of all groups by two-sided log-rank test.

Supplementary References

1. Vacchelli E, Ma Y, Baracco EE, et al. Chemotherapy-induced antitumor immunity requires formyl peptide receptor 1. *Science* 2015;350(6263):972-8

2. Chen DN, Song CG, Yu KD*, et al.* A Prospective Evaluation of the Association between a Single Nucleotide Polymorphism rs3775291 in Toll-Like Receptor 3 and Breast Cancer Relapse. *PLoS One* 2015;10(7):e0133184.

3. Castro FA, Forsti A, Buch S*, et al.* TLR-3 polymorphism is an independent prognostic marker for stage II colorectal cancer. *Eur J Cancer* 2011;47(8):1203-10.

4. Apetoh L, Ghiringhelli F, Tesniere A, et al. Toll-like receptor 4-dependent contribution of the immune system to anticancer chemotherapy and radiotherapy. *Nat Med* 2007;13(9):1050-9.

5. Tesniere A, Schlemmer F, Boige V, et al. Immunogenic death of colon cancer cells treated with oxaliplatin. *Oncogene* 2010;29(4):482-91.
